# Modification of the HeRO Graft Allowing Earlier Cannulation and Reduction in Catheter Dependent Days in Patients with End Stage Renal Disease: A Single Center Retrospective Review

**DOI:** 10.1155/2014/318629

**Published:** 2014-01-06

**Authors:** Deirdre Hart, Christie Gooden, L. S. Cummings, Brandt C. Wible, John Borsa, Henry Randall

**Affiliations:** ^1^General Surgery Department, University of Missouri-Kansas City School of Medicine, Kansas City, MO 64108, USA; ^2^Saint Luke's Hospital Transplant Specialists, Kansas City, MO 64111, USA; ^3^Section of Interventional Radiology, UMKC-SOM, Saint Luke's Hospital of Kansas City, 4401 Wornall Road, Kansas City, MO 64111, USA; ^4^Saint Luke's Hospital Interventional Radiology Department, Kansas City, MO 64111, USA

## Abstract

After creation of an arteriovenous fistula or placement of an arteriovenous graft, several weeks are required for maturation prior to first cannulation. Patients need an alternative way to receive hemodialysis during this time, frequently a catheter. After multiple failed access attempts, patients can run out of options and become catheter dependent. At our institution, we place HeRO grafts in eligible patients who have otherwise been told they would be catheter dependent for life. By combining the HeRO graft system with a Flixene graft, patients are able to remove catheters sooner or avoid placement as they can undergo cannulation for hemodialysis the next day. Utilizing this novel technique, twenty-one patients over a two-year period with various forms of central venous stenosis, catheter dependence, or failing existing arteriovenous access have been successfully converted to stable long term noncatheter based upper extremity access.

## 1. Introduction

According to the National Kidney and Urologic Diseases Information Clearinghouse; there were 398, 861 patients with end stage renal disease (ESRD) being treated with dialysis in 2009, and about 18% used a catheter for hemodialysis [1]. Once a patient is found to have central venous stenosis or occlusion, attempts at upper extremity long term hemodialysis access are often abandoned if it cannot be corrected [2]. As a result, these patients are relegated to lower extremity arteriovenous grafts or tunneled catheters for hemodialysis access. The HeRO graft was developed as an option for patients who are in this predicament.

**Figure 1:**
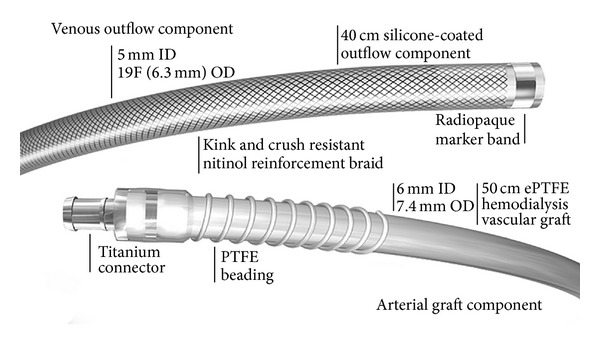
Used with the permission of CryoLife, Inc.

**Figure 2:**
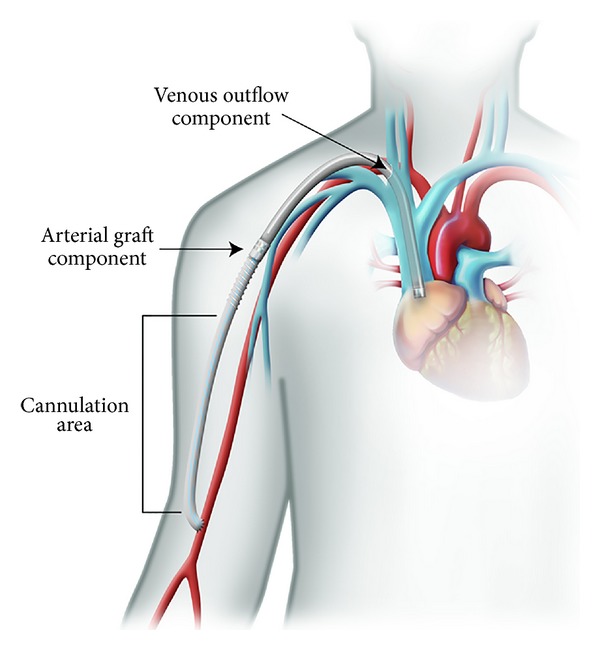
Used with the permission of CryoLife, Inc.

A HeRO (Hemodialysis Reliable Outflow) graft is made of a venous outflow component and an arterial graft component (Figure 1). It is FDA approved as a hemodialysis graft and is located completely subcutaneously. The 19F outflow component has a radiopaque tip to assist with placement. There is a titanium connector to connect the venous outflow to the arterial ePTFE graft in the deltopectoral groove (Figure 2).

## 2. Materials and Methods

In 2011, the Saint Luke's Hospital Transplant Specialists started the Dialysis Access Center consisting of two surgeons and four interventional radiologists. Several patients considered catheter dependent were referred to the Dialysis Access Center and the HeRO graft was offered as an option for this subset of patients. Since that time we have placed twenty-one HeRO grafts in twenty-one patients. Patients range in age from twenty-five to eighty-nine and have been on hemodialysis from two to twenty years, with a mean of 7.5 years on hemodialysis. Fifteen patients had HeRO grafts placed secondary to multiple failed access attempts secondary to central venous stenosis. Two were placed secondary to superior vena cava occlusion requiring recanalization. Two were used to rescue existing fistulas that were failing due to ipsilateral central stenosis by connecting the fistula to the HeRO outflow component. In addition, two had central stenosis with poor peripheral arterial systems requiring proximalization of arterial inflow during graft placement. The first four procedures were performed as per the FDA approved method. Subsequently two modifications were made to the originally prescribed procedure to make it better suited to our complex patient population (Table 1).

**Figure 3:**
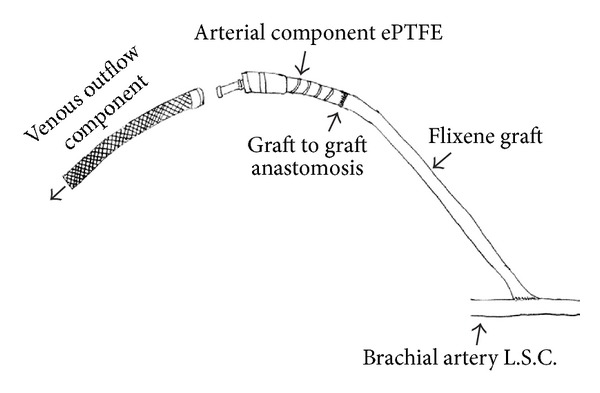


It was noted that the approved method required a two-week waiting period before cannulation of the ePTFE component. This method still required the use of a tunneled catheter in the interim period. In patients who have limited access or in whom infection is a concern, obtaining or having an additional catheter is often problematic. In an attempt to obviate the need for this two-week waiting period, a portion of the ePTFE arterial component was replaced with a Flixene graft. The APHECS II trial showed no complications cannulating the Flixene graft within 72 hours of placement for hemodialysis access [3]. At our institution, Flixene grafts are routinely used for standard AV graft formation and success has been had with cannulation as early as postoperative day one. Using this experience, the decision was made to transect the ePTFE just proximal to the titanium connector. The Flixene graft is then anastomosed to the remaining ePTFE in an end-to-end fashion with 5-O prolene suture. The Flixene graft is then anastomosed to the inflow artery (Figure 3).

This modification has allowed temporary catheters to be removed sooner, or in some cases, to be completely replaced by the HeRO; thereby negating the need for an accessory catheter.

The second modification involves protecting the hybrid anastomosis. The Flixene to ePTFE connection is often immersed in the tunnel. To prevent bleeding, BioGlue is used to seal the anastomosis. The remaining seventeen patients received a HeRO graft using this technique.

Given the complexity of these patients, selection and work-up is of utmost importance. The majority of our referrals come with no available previous work-up and are often from a distance away. Therefore, patients are often admitted to the hospital the day prior to surgery to complete this work-up. The work-up includes bilateral upper extremity central venograms to assess patency of the venous system. Patients with known or suspected peripheral arterial disease also undergo bilateral upper extremity arteriograms. These studies are performed by our interventional radiology colleagues. As the patients often have a complex medical history with multiple medical comorbidities, a cardiopulmonary work-up can also be completed during this preoperative admission.

After review of the venogram and/or arteriogram the upper extremity for implantation is chosen. If venoplasty with or without stenting of the central venous system, including recanalization of central vessels, is required, it is performed at this time. In order to facilitate access to stenosed or previously occluded central veins intraoperatively, a temporary dialysis catheter is often also placed during this time. This allows the venous outflow component of the HeRO graft to be placed over a guidewire without difficulty. During the operative placement of the venous outflow component of the HeRO graft the following day, a guidewire can be placed through the temporary catheter, which is then subsequently removed. The tract is serially dilated and the venous component is positioned using fluoroscopic guidance. Having these interventions performed prior to proceeding to the operating room allows delineation of venous anatomy, identification of trouble areas, and pretreatment of stenosis. This has translated into assurance that the HeRO graft could be placed prior to proceeding to the operating room and undertaking the additional risks of general anesthesia.

## 3. Results and Discussion

We have placed twenty-one HeRO grafts in twenty-one patients over two years. Complications have included one perioperative myocardial infarction, four infections that required excision (one primary HeRO graft infection and three secondary infections from other primary sites), and one dialysis-associated steal syndrome that required ligation (in a patient without known peripheral arterial disease) (Table 2). At the time of this paper, five HeRO grafts have been removed for the above mentioned causes, two have failed due to poor inflow, 4 patients have died of other causes with functioning HeRO grafts, and ten HeRO grafts continue to function for hemodialysis.

HeRO grafts patency rates are comparable to standard arteriovenous grafts [4], but in our experience they respond well to thrombectomy. To that end, patients need enough of an ejection fraction to maintain flow throughout the length of the access. When considering HeRO graft placement, the company recommends an ejection fraction over 20% [5].

Catheter dependence places patients at high risk for infections and potentially subsequent hospitalization [6]. Centers for Medicare and Medicaid Services recognizes this and has encouraged the reduction of the number of catheter dependent patients in order to save valuable healthcare dollars. To this end dialysis centers are now held accountable for their catheter rates and can be penalized monetarily [7]. HeRO grafts are a viable option for hemodialysis patients who are running out of access or who are already catheter dependent secondary to central venous stenosis or occlusion. By using a combined approach with interventional radiology, one can gain valuable information about a patient's vasculature prior to proceeding to the operating room. This allows maximal chances of success in placement of hemodialysis graft access and reduced catheter dependence.

## 4. Conclusion

Central venous stenosis, often from the over use of catheters, is a serious problem in the ESRD population leading to loss of access and catheter dependence. The HeRO graft represents an advance that can obviate the need for long term tunneled catheters. Our method allows for more expedited use of this system, reducing catheter dependent days and thus decreasing risk for infections and death. This is achieved by allowing cannulation on postoperative day one and using BioGlue to reduce bleeding at the hybrid anastomosis.

## Figures and Tables

**Table 1:** 

Pertinent patient comorbidities	Number of patients
Hypertension	18
Diabetes mellitus	12
Cardiac disease	11
Anemia	9
Dyslipidemia	9
Cerebrovascular accident	6
Thyroid disease	6
Obstructive sleep apnea	5
Asthma	4
Deep vein thrombosis	3
Gastroparesis	3
Hyperparathyroidism	3
Morbid obesity	3
Chronic obstructive pulmonary disease	2
Seizures	2
HIV/AIDS	1
Paraplegia	1
Parkinson's disease	1
Pulmonary hypertension	1

**Table 2:** 

Complication	Intervention	Patients
Perioperative myocardial infarction	Cardiac catheterization	1
Infection	Excision of graft	4
Dialysis-associated steal syndrome	Graft ligation	1
